# Uptake of SARS-CoV-2 vaccination among structurally-marginalized people who use drugs in Vancouver, Canada

**DOI:** 10.1038/s41598-023-44069-8

**Published:** 2023-10-20

**Authors:** Hudson Reddon, Brittany Barker, Sofia Bartlett, Ana Citlali Márquez, Inna Sekirov, Agatha Jassem, Muhammad Morshed, Ari Clemens, Phoenix Beck McGreevy, Kanna Hayashi, Kora DeBeck, Mel Krajden, M.-J. Milloy, Maria Eugenia Socías

**Affiliations:** 1 BC Centre on Substance Use, 400-1045 Howe St., Vancouver, BC V6Z 2A9 Canada; 2 Division of Social Medicine, Department of Medicine, University of British Columbia, 2775 Laurel St., Vancouver, BC V5Z 1M9 Canada; 3 Public Health Laboratory, BC Centre for Disease Control, Vancouver, BC V5Z 4R4 Canada; 4 Department of Pathology and Laboratory Medicine, University of British Columbia, Vancouver, BC V6T 1Z7 Canada; 5 Faculty of Health Sciences, Simon Fraser University, 8888 University Dr, Burnaby, BC V5A 1S6 Canada; 6 School of Public Policy, Simon Fraser University, 8888 University Dr, Burnaby, BC V5A 1S6 Canada

**Keywords:** Health services, Risk factors

## Abstract

We sought to evaluate the rates and predictors of SARS-CoV-2 vaccination among members of a structurally-marginalized population of people who use drugs (PWUD) during a targeted, community-wide, vaccination campaign in Vancouver, Canada. Interviewer-administered data were collected from study participants between June 2021 and March 2022. Generalized estimating equation analysis was used to identify factors associated with SARS-CoV-2 vaccine uptake, ascertained through a province-wide vaccine registry. Among 223 PWUD, 107 (48.0%) reported receipt of at least two SARS-CoV-2 vaccine doses at baseline and this increased to 151 (67.7%) by the end of the study period. Using social media as a source of vaccine information was negatively associated with SARS-CoV-2 vaccine uptake (Adjusted odds ratio [AOR] 0.27, 95% confidence interval [CI] 0.09–0.81) and HIV seropositivity (AOR 2.68, 95% CI 1.12–6.39) and older age (AOR 1.27, 95% CI 1.07–1.51) were positively associated with SARS-CoV-2 vaccine uptake. These findings suggest that the targeted vaccination campaign in Vancouver may be an effective model to promote SARS-CoV-2 vaccination in other jurisdictions. However, using social media as a source of vaccine information likely reduced SARS-CoV-2 vaccine uptake in PWUD arguing for further efforts to promote accessible and evidence-based vaccine information among marginalized populations.

## Introduction

People who use drugs (PWUD) and reside in urban settings experience multiple and intersecting socio-structural, behavioural, environmental, and biological risk factors for acquisition of SARS-CoV-2 and development of serious cases of coronavirus disease (COVID-19)^1–4^. Early investigations indicate that certain factors common among PWUD—including poverty, congregate living (e.g., shelters, correctional facilities), high-intensity drug use, racialization and associated comorbidities (e.g., HIV infection)—increase the risk of SARS-CoV-2 infection and COVID-19-related morbidity and mortality^1, 2, 4^. Previous studies from North America and Europe have estimated early wave COVID-19 prevalence rates as high as 36% among people who are homeless, an eight-fold increase in the odds of SARS-CoV-2 infection among people with a substance use disorder (SUD) and a two-fold increase in the hazard ratio of COVID-19 mortality among people living with HIV^1, 2, 4^. Given the vulnerability of PWUD, this population was identified as a priority group for SARS-CoV-2 vaccination and ongoing evaluations of the prevalence and predictors of SARS-CoV-2 vaccine uptake are needed to inform public health programs and promote up-to-date vaccination among this population^5, 6^.

Existing studies and public health surveillance data have found that the SARS-CoV-2 vaccine uptake among people who use drugs (PWUD) has ranged from 10 to 49% throughout the COVID-19 pandemic and was substantially lower than the uptake in the general population^3, 5–9^. Factors positively associated with vaccine uptake in these studies included prior history of SARS-CoV-2 diagnostic testing, opioid use and receipt of opioid agonist therapy, while female sex and high-frequency injection drug use were negatively associated with vaccine uptake^7^. Other studies have found that older age, food insecurity and increased concern about acquiring SARS-CoV-2 were associated with vaccine confidence^6, 8^. In contrast, access to smart phones, using social media as the primary source of COVID-19 information and exposure to COVID-19-related disinformation were independently associated with vaccine hesitancy^8^. Living with comorbidities has been linked to both vaccine hesitancy and vaccine confidence^6, 8^. The lower rates of SARS-CoV-2 vaccine uptake, and barriers to healthcare access among PWUD, highlight the need for targeted vaccination programs among this population^6, 8^.

In Vancouver, Canada, the Downtown Eastside (DTES) neighbourhood is home to a concentrated population of PWUD with high rates of socio-structural marginalization (e.g., homelessness and criminalization), medical comorbidities (e.g., mental health disorders and infectious diseases), and substance use^10, 11^. SARS-CoV-2 vaccines first became available in December of 2020 and from January 2021 to April 2022, residents in the DTES were prioritized for a targeted vaccination program that included pop-up vaccination clinics, street outreach, and small incentives to reduce the structural barriers to vaccine access among PWUD^12^. The vaccine program involved offering a minimum of three clinics per week in various locations (e.g., parks, parking lots, shelters) and incentives offered included small compensation such as gift cards, coffee, snacks and cash ($5 CAD)^13^. The four vaccines available included Pfizer and Moderna mRNA vaccines, the AstraZeneca viral vector vaccine and the Novavax protein-based vaccine. The majority of the population received mRNA vaccines and individuals were generally not permitted to select which vaccine they received^14^. COVID-19 testing was only available in healthcare settings until January 2022 when home testing kits became available, yet were in limited supply^14, 15^. PWUD are typically underrepresented in primary health care settings due to drug use stigma and discrimination, costs, and resources associated with healthcare access (e.g., transportation, access to booking systems) and medical mistrust^16–18^. Medical mistrust in particular has been linked with vaccine hesitancy in this population, and PWUD historically have lower uptake of other recommended vaccines, such as hepatitis A, hepatitis B and influenza, among others^17, 19^.

Despite these barriers and structural marginalization of PWUD, we are unaware of any studies that have longitudinally evaluated SARS-CoV-2 vaccine uptake of among PWUD during the COVID-19 pandemic. Given that that PWUD have also been found to be at increased risk of breakthrough SARS-CoV-2 infections following vaccination due to comorbid health conditions and socio-economic disadvantage^20^, identifying predictors of vaccine uptake and promoting up-to-date vaccination among this population is an important public health objective^20^. We therefore undertook the present study to evaluate the prevalence and predictors of SARS-CoV-2 vaccine uptake among a prospective cohort of PWUD in the context of a targeted vaccination program in Vancouver, Canada.

## Methods

Three open community-recruited prospective cohort studies provided the data for this study. These cohorts include the At-Risk Youth Study (ARYS), the Vancouver Injection Drug Users Study (VIDUS), and the AIDS Care Cohort to evaluate Exposure to Survival Services (ACCESS), which have been described in detail previously^21, 22^. Cohort participants were recruited through extensive street outreach and self-referral from the Downtown Eastside and Downtown South neighbourhoods of Vancouver, Canada. The recruitment, follow-up, and data collection procedures have been harmonized across these cohorts to facilitate pooled analyses^21, 22^.

Eligibility criteria for the three cohorts included residing in the Greater Vancouver Regional District, using drugs (other than or in addition to cannabis) in the previous month and providing written informed consent. The ARYS cohort includes youth aged 14–26 years who are without stable housing or report accessing street-based youth services in the past 6 months. VIDUS includes adults who report injection drug use in the month prior to enrolment and are HIV seronegative, and ACCESS includes adults who are living with HIV and report using drugs (other than or in addition to cannabis) in the month prior to enrolment. Informed consent from a parent or legal guardian was obtained for ARYS participants younger than 18 years of age. In June 2021, a convenience sample of 275 participants recruited from these cohorts were invited to enroll in the present study. All participants from the three cohorts were eligible to participate. After enrolment, participants completed four study visits that were each 2 months apart. Each visit involved completing an interviewer-administered questionnaire that collected data including socio-demographic information, substance use behaviours, attitudes towards COVID-19, and attitudes towards and uptake of SARS-CoV-2 vaccines (Supplementary Table 1). They also provided dried blood spot samples for serological analyses. At each study visit, participants were remunerated $40 CAD for their time. The Providence Health Care/University of British Columbia Research Ethics Board has provided ethical approval for this study and has also approved the ARYS, VIDUS and ACCESS studies on an annual basis. All methods were carried out in accordance with the relevant guidelines and regulations.

The study period for the present analysis was from June 2021 to March 2022 and consenting participants completed four study visits during this time that were each 2 months apart. The analytical sample included all consenting participants who completed a baseline visit between June and August 2021. The primary outcome of interest was time to receipt of at least two COVID-19 vaccine doses. The number of COVID-19 vaccination doses received and the date of vaccination, was ascertained through a confidential linkage with the British Columbia Centre for Disease Control province-wide vaccine registry. Personal health numbers were used to conduct this linkage and all study participants provided informed consent to link their vaccination records with data collected for the present study. Vaccination rates in the present study were compared to provincial rates using publicly available records published by the government of Canada^9^.

For this analysis, we estimated the longitudinal relationships between sociodemographic, behavioural, social-structural and clinical factors with receipt of two SARS-CoV-2 vaccine doses. Explanatory variables included: sex at birth (female vs. male); age (continuous, in 5-year increments); race/ethnicity (White vs. Black, Indigenous and People of Colour (BIPOC)); education (< secondary school/< 12 years of education vs. ≥ secondary school/≥ 12 years of education); congregate living (i.e., shelter, single room occupancy hotel, correctional facility vs. no); homelessness (yes vs. no); self-reported suspected COVID-19 infection in the last 2 months (yes vs. no); previous positive COVID-19 test result (yes vs. negative result or never tested); use of social media for vaccine information (yes vs. no); non-injection drug use (yes vs. no); injection drug use (yes vs. no); and HIV serostatus (positive vs. negative). Among participants living with HIV, a confidential data linkage with the province’s centralized ART dispensary and HIV treatment registry (the British Columbia Centre for Excellence in HIV/AIDS’ Drug Treatment Program) provided a complete retrospective and prospective clinical profile including each participant’s VL and CD4+ cell count tests and details of all of their ART dispensations. All participants included in this study provided written informed consent to this linkage. Each behavioural variable referred to the previous 2-months period and variable definitions were consistent with previous studies^22^.

The baseline characteristics of the study sample, stratified by COVID-19 vaccination status, were analyzed using the Chi square test for binary variables and the Wilcoxon’s rank sum test for continuous variables. Generalized estimating equations (GEE) models were used to analyze the factors associated with vaccine uptake over the study period. By using an exchangeable correlation structure, this method facilitated the identification of factors associated with the outcome over the entire study period and calculated standard errors while adjusting for multiple observations from each individual. Since GEE models are a form of marginal longitudinal analysis, they have been used to analyze data including repeated measures, such as longitudinal cohorts^23^. As a first step, unadjusted models were built to analyze the association between each predictor variable and the outcome (receipt of two SARS-CoV-2 vaccine doses). The multivariable model included all explanatory variables.

We also conducted a secondary analysis using extended Cox regression models with time-updated covariates to identify factors associated with time to vaccine uptake over the follow-up period among participants who had less than two SARS-CoV-2 vaccine doses at baseline. We first built unadjusted models to analyze the association between each predictor variable and the outcome (time to receipt of two SARS-CoV-2 vaccine doses). The multivariable model included all explanatory variables that were associated with the outcome at the level *P* < 0.05 in the unadjusted models. Participants who reported a positive COVID-19 test in the past 2 months were removed from the vaccine uptake analyses (GEE and Cox regression models) for the 6-month period following their positive test, since vaccination guidelines recommended delaying primary series vaccine receipt for 6 months following SARS-CoV-2 infection^24^. All statistical analyses were performed using SPSS (version 27; IBM Corporation, New York, NY). All *p* values are two-sided, with a significance threshold of *P* < 0.05.

## Results

**Figure 1:**
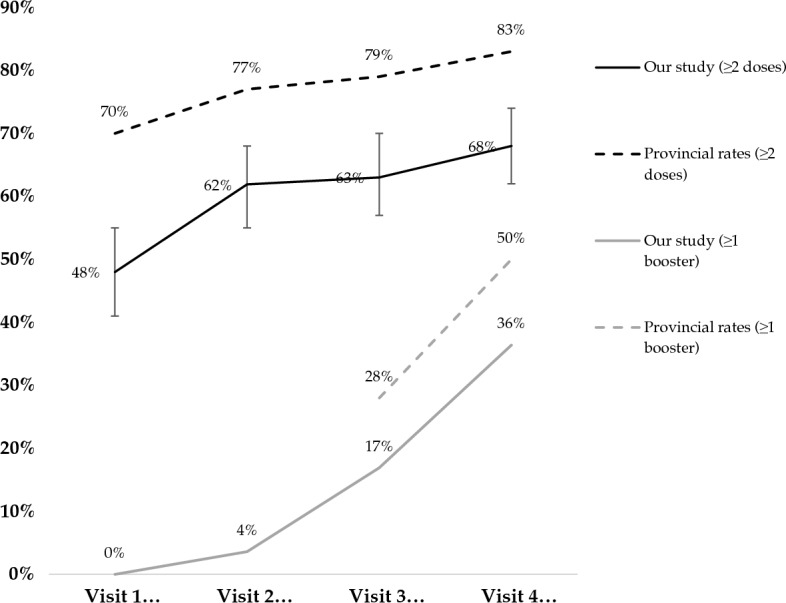
Prevalence of SARS-CoV-2 vaccination over study follow-up compared to provincial rates in British Columbia.

A total of 275 participants were enrolled in this study between June and August 2021, and 223 had a valid personal health number to facilitate linkage with the provincial vaccine registry. Of the 223 participants, 76 (34.1%) were female, 117 (52.5%) self-identified as White and the median age at baseline was 49.0 years (interquartile range [IQR] 35.0–57.0). Of the 93 (41.7%) participants living with HIV, 92 (98.9%) were linked to HIV care, 91 (97.8%) had initiated antiretroviral therapy, and 78 (83.9%) were virally suppressed (≤ 1000 copies/mL)^25^. There were 11 participants living with HIV who were moderately to severely immunosuppressed (CD4 cell count < 200 cells/mm^3^) and they were excluded from the analysis since there are unique guidelines for SARS-CoV-2 vaccination (three-dose primary series) among people who meet this threshold of immunosuppression^26^. The majority of the participants completed all four study visits (N = 165, 74.0%) and the follow-up rate at each study visit was over 80%. At each of the four study visits, the number of participants who reported receiving at least two SARS-CoV-2 vaccine doses was 107 (48.0%), 138 (61.9%), 140 (62.8%) and 151 (67.7%), respectively (Fig. 1). There were no participants who received a SARS-CoV-2 vaccine booster dose at baseline as it was not available in the study setting at the time. However, 2 (0.9%), 19 (8.5%) and 62 (27.8%) participants reported receiving at least one booster dose over the subsequent three study visits. SARS-CoV-2 vaccine uptake relative to the provincial rates in British Columbia is shown in Fig. 1^9^. The confidence interval estimates for the rates of vaccine uptake indicate that there was a statistically significant difference in the rates of vaccine uptake between our study sample and the general population in British Columbia (Fig. 1).

At baseline, 144 (64.6%) participants reported living in a collective dwelling, 97 (43.5%) reported injection drug use, 40 (17.9%) reported a suspected COVID-19 infection in the past 2 months and 32 (14.3%) reported using social media as a source of information for SARS-CoV-2 vaccination decisions. Of the vaccinations doses received by study participants, the most common sites of vaccination were vaccination events/pop-ups in parking lots, parks or drive-throughs (48.5%), doctor’s offices (16.6%) and community health centres (10.8%). The participants also reported strong adherence to COVID-19 public health measures with 128 (57.4%) participants reporting masking and physical distancing always (100% of the time) or usually (> 75% of the time). The association between adherence to these public health measures and vaccine uptake over follow-up was not statistically significant (P = 0.597). The characteristics of the study sample stratified by vaccination status at baseline are shown in Table 1.

**Table 1:** Baseline characteristics of the study sample stratified by COVID-19 vaccination status among people who use unregulated drugs in Vancouver, Canada (n = 223).

Characteristic	Total n (%)	COVID-19 vaccination status			*P-*value
		Unvaccinated n (%) 65 (29.1%)	One dose n (%) 51 (22.9%)	Two doses n (%) 107 (48.0%)	
Age					
Median	49.0	38.0	46.0	54.0	** < 0.001**
IQR	(35.0–57.0)	(30.0–53.0)	(30.0–55.0)	(44.0–59.0)	
Sex at birth					
Male	147 (65.9)	40 (61.5)	32 (62.7)	75 (70.1)	0.446
Female	76 (34.1)	25 (31.5)	19 (37.3)	76 (34.1)	
Ethnicity*					
Non-White	105 (47.1)	33 (51.6)	22 (43.1)	62 (57.9)	0.214
White	117 (52.5)	31 (48.4)	29 (56.9)	45 (42.1)	
Highest level of education					
< High school	105 (47.1)	28 (43.1)	23 (45.1)	54 (50.5)	0.609
≥ High school	118 (52.9)	37 (56.9)	28 (54.9)	53 (49.5)	
Congregate living**					
Yes	144 (64.6)	39 (60.0)	33 (64.7)	72 (67.3)	0.625
No	79 (35.4)	26 (40.0)	18 (35.3)	35 (32.7)	
Homelessness					
Yes	21 (9.4)	11 (16.9)	2 (3.9)	8 (7.5)	**0.037**
No	202 (90.6)	54 (83.1)	49 (96.1)	202 (90.6)	
Suspected COVID-19 infection^a^					
Yes	40 (17.9)	15 (24.2)	9 (18.4)	16 (15.4)	0.369
No	175 (78.5)	47 (75.8)	40 (81.6)	88 (84.6)	
Received + COVID-19 test^a^					
Yes	16 (7.2)	5 (16.1)	3 (7.7)	8 (9.8)	0.492
No or never tested	136 (61.0)	26 (83.9)	36 (92.3)	74 (90.2)	
Using social media for vaccine information					
Yes	52 (23.3)	28 (46.7)	9 (18.0)	15 (14.0)	** < 0.001**
No	165 (74.0)	32 (53.3)	41 (82.0)	92 (86.0)	
Non-injection drug use^a^					
Yes	129 (57.8)	40 (61.5)	33 (64.7)	56 (52.3)	0.262
No	94 (42.2)	25 (38.5)	18 (35.3)	51 (47.7)	
Injection drug use^a^					
Yes	97 (43.5)	26 (40.0)	27 (52.9)	44 (41.1)	0.298
No	126 (56.5)	39 (60.0)	24 (47.1)	63 (58.9)	
HIV seropositive					
Yes	93 (41.7)	18 (27.7)	23 (45.1)	52 (48.6)	**0.023**
No	130 (58.3)	47 (72.3)	28 (54.9)	55 (51.4)	

*IQR* interquartile range.

^a^Refers to activities in the 2 months prior to the interview.

*Participants were asked to select all ethnic groups they identify with, not all cells may add up to 223 as participants may choose not to answer some questions.

**Collective dwelling is defined as living in a SROs, shelters, hotels/motels, correctional facilities, groups homes, dorms, emergency lodging. Bold text refers to *P*-values < 0.05.

The unadjusted and adjusted GEE analysis of factors associated with SARS-CoV-2 vaccine uptake (at least two doses) is shown in Table 2. In the adjusted analysis, age (OR 1.27, 95% confidence interval [CI] 1.07–1.51; P = 0.006) and HIV seropositivity (OR 2.68, 95% CI 1.12–6.39; P = 0.027) were significantly associated with increased odds of SARS-CoV-2 vaccine uptake, while using social media as a source of vaccine information was significantly associated with lower odds of SARS-CoV-2 vaccine uptake (OR 0.27, 95% CI 0.09–0.81; P = 0.019). The interaction between the use of social and time was not statistically significant (OR 0.48, 95% CI 0.14–1.62; P = 0.237), indicating that the association between the use of social media and vaccine uptake did not vary significantly during the study period.

**Table 2:** Unadjusted and adjusted generalized estimating equation analysis of factors associated with SARS-CoV-2 vaccine uptake (at least two doses).

Characteristic	Unadjusted			Adjusted	
	OR (95% CI)	*P-*value		OR (95% CI)	*P-*value
Age					
(Per 5 years older)	1.39 (1.20, 1.60)	** < 0.001**		1.27 (1.07, 1.51)	**0.006**
Sex at birth					
(Male vs. female)	0.73 (0.37, 1.42)	0.355		0.97 (0.45, 2.11)	0.946
Ethnicity					
(White vs. non-white)	0.72 (0.38, 1.39)	0.330		0.73 (0.32, 1.66)	0.456
Education					
(< High school vs. ≥ high school)	1.23 (0.64, 2.35)	0.534		1.71 (0.83, 3.56)	0.148
Congregate living**					
(Yes vs. no)	0.91 (0.46, 1.81)	0.792		0.72 (0.30, 1.74)	0.468
Homelessness					
(Yes vs. no)	0.37 (0.16, 0.85)	**0.019**	(− 1.21, 0.67)	0.67 (0.26, 1.70)	0.398
Suspected COVID-19					
(Yes vs. no)	0.43 (0.22, 0.87)	**0.018**		0.60 (0.19, 1.87)	0.376
Received + COVID-19 test^a^					
(Yes vs. no or never tested)	0.44 (0.21, 0.93)	**0.031**		0.63 (0.17, 2.36)	0.491
Using social media for vaccine information					
(Yes vs. no)	0.24 (0.10, 0.59)	**0.002**		0.27 (0.09, 0.81)	**0.019**
Non-injection drug use^a^					
(Yes vs. no)	0.50 (0.27, 0.91)	**0.024**		0.76 (0.38, 1.51)	0.436
Injection drug use^a^					
(Yes vs. no)	0.54 (0.27, 1.11)	0.095		0.68 (0.30, 1.52)	0.344
HIV seropositive					
(Yes vs. no)	3.86 (1.87, 7.98)	** < 0.001**		2.68 (1.12, 6.39)	**0.027**

*OR* hazard ratio, *CI* confidence interval.

^a^Refers to activities in the 2 months prior to the follow-up interview.

**Congregate living is defined as living in a SROs, shelters, hotels/motels, correctional facilities, groups homes, dorms, emergency lodging, bold text refers to *P*-values < 0.05.

The unadjusted and adjusted hazard ratios for SARS-CoV-2 vaccine uptake (at least two doses) among the 116 participants with less than two SARS-CoV-2 vaccine doses at baseline are presented in Table 3. In the adjusted analysis, only higher levels of education (HR 1.77, 95% CI 1.12–1.79; P = 0.014) and using social media information to make decisions about vaccination (HR 0.42, 95% CI 0.18–0.97; P = 0.043) were associated with lower rates of SARS-CoV-2 vaccine uptake.

**Table 3:** Bivariable and multivariable Cox regression analysis of factors associated with time to SARS-CoV-2 vaccine uptake.

Characteristic	Unadjusted			Adjusted	
	HR (95% CI)	*P-*value		HR (95% CI)	*P-*value
Age					
(Per 5 years older)	1.08 (0.99, 1.16)	0.050			
Sex at birth					
(Male vs. female)	1.36 (0.92, 2.00)	0.125			
Ethnicity					
(White vs. non-white)	1.30 (0.88, 1.92)	0.194			
Education					
(< High school vs. ≥ high school)	1.55 (1.03, 2.33)	**0.034**		1.77 (1.12, 1.79)	**0.014**
Congregate living**					
(Yes vs. no)	0.72 (0.48, 1.10)	0.130			
Homelessness					
(Yes vs. no)	0.39 (0.18, 0.84)	**0.016**	(− 1.21, 0.67)	0.52 (0.23, 1.15)	0.106
Suspected COVID-19					
(Yes vs. no)	0.46 (0.14, 1.46)	0.188			
Received + COVID-19 test^a^					
(Yes vs. no or never tested)	0.61 (0.08, 4.55)	0.631			
Using social media for vaccine information					
(Yes vs. no)	0.35 (0.15, 0.79)	**0.012**		0.42 (0.18, 0.97)	**0.043**
Non-injection drug use^a^					
(Yes vs. no)	0.92 (0.60, 1.41)	0.694			
Injection drug use^a^					
(Yes vs. no)	1.06 (0.69, 1.63)	0.786			
HIV seropositive					
(Yes vs. no)	1.55 (1.04, 2.32)	**0.032**		1.09 (0.66, 1.81)	0.744

*HR* hazard ratio, *CI* confidence interval.

^a^Refers to activities in the 2 months prior to the follow-up interview.

**Congregate living is defined as living in a SROs, shelters, hotels/motels, correctional facilities, groups homes, dorms, emergency lodging, bold text refers to *P*-values < 0.05.

Among participants who were vaccinated or planning to receive a SARS-CoV-2 vaccine during the study period, the three most common reasons cited for vaccination were concerns about acquiring COVID-19 (46.2%), concerns about transmitting COVID-19 (42.4%) and confidence in vaccine safety (25.3%). Among those who were opposed or undecided about receiving a SARS-CoV-2 vaccine, the three most common reasons were concerns about vaccine side effects (48.4%), concerns about vaccine safety (25.8%) and government mistrust (9.7%). Among the 36 participants citing these reasons for vaccine hesitancy, the rates of vaccine uptake were significantly lower than among participants who did not report these concerns (P < 0.001). Only 6 (16.7%) of these participants received two SARS-CoV-2 vaccine doses and only one (2.8%) participant received a booster dose. Older participants were more likely to report concerns about vaccine side-effects (P = 0.027) as a reason for vaccine hesitancy while younger participants were more likely to report safety concerns (P = 0.037) as a reason for vaccine hesitancy. Older participants were more likely to report concerns about acquiring COVID-19 (P < 0.001) and confidence in vaccine safety (P < 0.001) as sources of vaccine confidence. Younger participants were significantly more likely to report using social media as a source of vaccine information (P = 0.002).

## Discussion

The objectives of the present study were to investigate the prevalence rates and predictors of SARS-CoV-2 vaccine uptake among a prospective cohort of PWUD. We observed that 48% of the participants received at least two SARS-CoV-2 vaccine doses prior to baseline (September 2021) and by the end of the study period (March 2022), 68% of the participants had received at least two SARS-CoV-2 vaccine doses and 21.5% had received three SARS-CoV-2 vaccine doses. In the multivariable analysis, using social media as a source of vaccine information was associated with a 73% decrease in the likelihood of vaccine uptake (i.e., people who did not use social media as a source of COVID-19 vaccine information had three times [1/0.27 = 3.70) the odds of being vaccinated compared to people who did use social media as a source COVID-19 vaccine information. People living with HIV and people of older age were significantly more likely to be vaccinated. In the secondary analysis of people who received less than two SARS-CoV-2 vaccines at baseline, using social media as a source of vaccine information, lower levels of education and younger age were associated with slower rates of vaccine uptake. The most common motivations for vaccine uptake were concerns about acquiring or transmitting COVID-19, while concerns about vaccine side effects and safety were the most frequently reported reasons for vaccine hesitancy.

To our knowledge, this is one of the first studies to longitudinally evaluate SARS-CoV-2 vaccine uptake among PWUD^27^. While the vaccination rates we observed among PWUD were 22 percentage points lower than those observed among the general population in British Columbia at baseline (48% vs. 70%), this discrepancy decreased to 15 percentage points by the end of the study period (68% vs. 83%). This difference is substantially smaller than what was observed in a 2021 study in the US–Mexico border region, where only 9% of people who inject drugs (PWID) received at least one dose of COVID-19 vaccine compared to 59% in the general population^8, 28^. It is important to note that the low prevalence of vaccine uptake in this setting may be attributed to low vaccine availability and vaccine hesitancy as well as the lack of a targeted vaccine program as occurred in the current study. A cross-sectional study conducted in 2021 among PWUD found rates of vaccine uptake (at least two doses) of 30% among PWID in Australia, which were significantly lower than prevalence rates in most Australian states and territories among the general population^7^. Given that the rates of vaccine uptake in our study were greater than other populations of PWUD, the targeted vaccine program implemented in the DTES may have been effective in promoting vaccine uptake by reducing barriers to SARS-CoV-2 vaccine access among PWUD^12^. However, these trends should be interpreted with caution as the impacts of this intervention were not directly compared in the same population. It is also possible that other factors that may have facilitated vaccine uptake in this setting, including a strong history of healthcare outreach in the Downtown Eastside community of Vancouver, such as needle exchange programs and seek, test and treat campaigns for HIV prevention^29^. Lower rates of criminalization based on the legalization and regulation of cannabis use and decriminalization of personal drug possession may have also reduced barriers and experiences of stigma associated with healthcare access among PWUD and thereby facilitated vaccine uptake^30, 31^. Differences in healthcare access (e.g., outreach, financial barriers) and structural factors (e.g., criminalization of substance use, substance use stigma) have been associated with vaccine hesitancy in previous studies and may contribute to the variability in vaccination uptake across jurisdictions among PWUD^17, 32, 33^. Healthcare access may have contributed to our observation that people living with HIV were more likely to have received two SARS-CoV-2 vaccine doses. People living with HIV may have increased contact with health services for HIV care, which could have led to increased access to SARS-CoV-2 vaccines.

It is also important to acknowledge that the emergence of SARS-CoV-2 variants and vaccine guidelines may have contributed to the trends vaccine uptake among the study sample^15^. The DTES vaccination strategy offered vaccines to all members of the community without restrictions on age or health status. In contrast, the general population offered staggered doses to different demographics based on age and vulnerability to illness^9, 12, 26^. This may explain the increased rates of vaccine uptake in the study sample between visits one and two compared to the general population. It is possible that vaccination may have approached a plateau among DTES residents earlier based on the vaccination campaign, and then increased in parallel with provincial rates in British Columbia where a staggered vaccination strategy was applied^9, 26^. While the Delta variant was the predominant variant in Canada from July to October of 2021, the Omicron variant, characterized by increased transmissibility and immune escape, became the dominant variant from November 2021 until the end of the study period (March 2022). The National Advisory Committee on Immunization (NACI) guidelines recommend receiving the primary series doses 8 weeks apart and initially recommended receiving booster doses 6 months after completion of the primary vaccination series, yet this interval was reduced to 3 months with the emergence of the Omicron variant in late November of 2021^34^. These guideline changes and increased public health messaging supporting vaccination may explain the sharp increases in booster dose uptake that we observed between study visit three (November 2021 to January 2022) and four (January to March 2022) among our sample and the general population in British Columbia^35^.

Existing evidence has shown that the majority of PWUD are willing to receive SARS-CoV-2 vaccines, suggesting that the low uptake in many settings reflects individual- (e.g., substance use, mental health comorbidities) and structural-level barriers (e.g., socioeconomic deprivation, homelessness, stigma) rather than vaccine hesitancy^8, 36^. The targeted low-barrier vaccine program in our study setting included drop-in vaccination events in community settings (e.g., parks, parking lots, street corners near the neighbourhood’s open drug market) and active outreach to shelters, single room occupancy hotels and people experiencing homelessness^12^. These services were designed to reduce structural barriers to vaccine access such as transportation, cost, and booking systems, as well as reduce the experiences of stigma and discrimination often experienced by PWUD in traditional medical settings^7, 33^. Since governmental and medical mistrust have been associated with vaccine hesitancy among PWUD, drug user and peer-led organizations were involved in the planning and delivery of multiple vaccination events to promote trust among community members^12, 17^. Other jurisdictions that have implemented SARS-CoV-2 vaccination programs through peer-based or harm reduction drop-in and outreach have been able to achieve vaccine uptake rates among PWID similar or equivalent to those observed in the general population^7^. Including people with lived/living experience of substance use (i.e., peers) in the planning and delivery of healthcare services has been shown to increase access and improve patient outcomes among PWUD^37, 38^. These results, in conjunction with our findings, support the use of similar strategies among members of structurally-marginalized populations to promote up-to-date SARS-CoV-2 vaccination as new variants emerge and additional vaccine doses are required to curb coronavirus-related morbidity and mortality. The success of this targeted vaccine outreach model may also be applicable to promote the uptake of seasonal vaccinations for other pathogens, such as influenza, among structurally marginalized populations.

Exposure to COVID-19 disinformation via social media has been linked to vaccine hesitancy among the general population and PWUD^8^. In this study, we extend these findings by showing that accessing SARS-CoV-2 information via social media was significantly associated with lower rates of vaccine uptake, and at least a high-school education was significantly associated with increased vaccine uptake. Seeking health information through social media may limit access to evidence-based information and increase exposure to COVID-19 disinformation that undermines vaccine uptake^8, 39^. This may have been particularly relevant for young adults in this study, as younger participants were more likely to report using social media as a source of vaccine information and were also more likely to report safety concerns as a primary reason for vaccine hesitancy. Although the associations between use of social media and education with vaccine uptake have been observed in existing studies^40, 41^, these findings are important to describe given the social and structural barriers experienced by people who use drugs. For instance, this population may have limited access to technological devices required for social media activity (e.g., smartphones) due to high rates of material insecurity and socioeconomic marginalization^42^. Demonstrating an association between social media exposure and vaccine uptake would indicate that this is an important method of communicating health and medical information during public health emergencies, even among populations who may have limited access to these social media platforms. In addition, the association between education and vaccine uptake may differ among populations with different variation in educational attainment. Among the general population, less than university education has been associated with vaccine hesitancy whereas less than high school education (12 years) was associated with vaccine hesitancy in the present study^40^. These findings may inform educational interventions tailored to distinct populations such as PWUD, which have been found to effectively promote vaccine uptake^43, 44^.

The preference for accessing alternative information sources rather than traditional medical advice among PWUD may stem from experiences of stigma and discrimination among PWUD in clinical settings^33, 45^. These experiences, in addition to the criminalization of substance use, have been found to contribute to medical and governmental mistrust, as well as limit engagement with health and harm reduction services^18, 46, 47^. We also found that the three most common reasons for vaccine hesitancy among in this study were concerns about vaccine side effects, concerns about vaccine safety and government mistrust. The same three reasons were also the most common reasons for vaccine hesitancy among the general population from low- middle- and high-income countries^48^. These findings reinforce the need to establish effective methods of promoting evidence-based vaccine information in accessible language and formats to the general population, as well as among marginalized populations such as PWUD^7, 8^. Other experts have suggested that vaccine information and interventions should be delivered by trusted sources of health information (e.g., peers, harm reduction staff, outreach workers) or community-based organizations to promote acceptability and confidence among PWUD^8, 49^. Our results support these findings and suggest the need to build collaborative and trusting relationships with PWUD and peer-based organizations, so that evidence-based vaccine information is accessible to people of diverse educational backgrounds and the exposure to disinformation via social media is limited.

### Limitations

This study has some limitations. Study variables were collected by self-report, which may have introduced socially desirable reporting and recall error. However, measuring substance use and other stigmatized behaviours among PWUD has been shown to provide valid and reliable measurements^50^. Since the ARYS, VIDUS and ACCESS cohorts are not random samples, these findings may not be generalizable to other PWUD. In addition, these cohort participants engage in regular contact with study and healthcare personnel and as a result, may display higher rates SARS-CoV-2 vaccination than other PWUD. Residual confounding may have influenced the results since this is an observational study. For 52 participants (18.9% of the original study sample), the identifiers provided were not sufficient to link personal health numbers and consequently, vaccination status and dates of vaccine administration were not available. It is possible that the associations we observed may have been influenced by this missing data. However, these non-linked participants did not show differences in sex, ethnicity, education, homelessness, non-injection or injection use and use of social media to make decisions about vaccination, relative to participants with linked data (P > 0.05). Participants with missing personal health numbers were more likely to be of younger age and HIV seronegative (P < 0.05). The data collection instrument did not capture the specific social media platform used to make decisions about SARS-CoV-2 vaccination. Since the recall period for suspected COVID-19 infection and COVID-19 testing was in the last 2 months, we did not capture SARS-CoV-2 infections that occurred in the full 6-month period prior to the start of the study period. This could have led to an underestimation of vaccine uptake since the portion of the sample who did receive a positive test were not eligible for vaccination. However, this may have had a modest impact on the results as a small proportion of the sample reported receiving a positive test at baseline (n = 16, 7.2%). Moreover, structural and behavioural barriers to testing in healthcare settings and acquiring home testing kits may have led to an underestimate of COVID-19 prevalence in the study sample. Lastly, using the general population from British Columbia as a comparison for vaccine uptake is limited in that there are many important differences between this population and PWUD that are likely to impact the uptake of SARS-CoV-2 vaccines. However, the reason for including the information from the general population was not to use them as a direct comparison, but rather to identify differences in vaccination rates experienced by PWUD based on structural and behavioural barriers to healthcare and vaccine access, even in the presence of an aggressive and targeted vaccination program designed to reduce barriers to vaccine uptake.

### Public health implications

In summary, we found that SARS-CoV-2 vaccine uptake among PWUD lagged behind rates in the general population, although this disparity decreased from 22 to 15 percentage points over the study period. This suggests that the targeted vaccination efforts among marginalized populations in our study setting were effective at promoting vaccine uptake to levels closer to those observed in the general population. We also found that using social media as a source of vaccination information was associated with lower rates of SARS-CoV-2 vaccine uptake and higher levels of education were associated with faster rates of vaccine uptake. These findings, in conjunction with other evidence, support the benefits of targeted vaccination efforts as well the expansion of informational campaigns that are co-designed with peer groups and community organizations to promote evidence-based vaccine information and facilitate informed vaccine decision-making.

### Supplementary Information

Supplementary Table 1.

## Data Availability

The datasets generated during and/or analyzed during the current study are not available due to the ethical agreements of the parent cohorts. Please contact M-J Milloy (bccsu-mjm@bccsu.ubc.ca) regarding access to the datasets generated and/or analysed for this study.
