# Supplementary material for: Uptake of SARS-CoV-2 vaccination among structurally-marginalized people who use drugs in Vancouver, Canada
Source: Sci Rep. 2023 Oct 20;13:17930. doi: 10.1038/s41598-023-44069-8 (PMC10589278; doi:10.1038/s41598-023-44069-8)
Supplement: Supplementary file 1 — Supplementary Table 1. [file 41598_2023_44069_MOESM1_ESM.pdf]

Supplementary Table 1. Questionnaire items collected by study interviewers.

	**Core Data Elements**	
4	Date of interview	______________ (DD-MM-YYYY)
5	What is your age? (years)	______________ ((YY))
6	What was your assigned sex at birth?	◯ MALE<br>◯ FEMALE<br>◯ PREFER TO SELF-DESCRIBE<br>◯ PREFER NOT TO ANSWER
11	How would you describe your ethnicity or race? [SELECT ALL THAT APPLY] If you are an Indigenous person (answered YES above), select any other that apply.	☐ WHITE<br>☐ SOUTH ASIAN<br>☐ CHINESE<br>☐ BLACK<br>☐ FILIPINO<br>☐ LATIN AMERICAN<br>☐ ARAB<br>☐ SOUTHEAST ASIAN<br>☐ WEST ASIAN<br>☐ KOREAN<br>☐ JAPANESE<br>☐ PREFER TO SELF-DESCRIBE<br>☐ PREFER NOT TO ANSWER
13	What is the highest level of education you have completed?	◯ LESS THAN HIGH SCHOOL GRADUATION<br>◯ HIGH SCHOOL GRADUATION<br>◯ TRADE CERTIFICATE, VOCATIONAL SCHOOL, OR APPRENTICESHIP TRAINING<br>◯ NON-UNIVERSITY CERTIFICATE OR DIPLOMA FROM A COMMUNITY COLLEGE, CEGEP<br>◯ UNIVERSITY BACHELOR'S DEGREE<br>◯ UNIVERSITY GRADUATE DEGREE (SUCH AS A MASTERS [illegible] DOCTORATE)<br>◯ PREFER NOT TO ANSWER
77	Have you been homeless in the last 2 months?	◯ YES<br>◯ NO
	**COVID-19 History**	
210	In the last 2 months, do you think you have had COVID-19?	◯ NO<br>◯ YES<br>◯ DON'T KNOW<br>◯ PREFER NOT TO ANSWER
215	In the last 2 months, have you ever been tested for an active COVID-19 infection (using nasopharyngeal/throat swab, saliva or gargle test)?	◯ NO<br>◯ YES<br>◯ DON'T KNOW<br>◯ PREFER NOT TO ANSWER
218	In the last 2 months, have you tested positive for COVID-19 on a test (nasopharyngeal/throat swab, saliva or gargle test)	◯ NO<br>◯ YES<br>◯ DON'T KNOW

78 What type of place are you living in now?

- ◯ APARTMENT
- ◯ ROOM IN HOTEL
- ◯ HOUSE
- ◯ SHELTER
- ◯ NFA/STREET
- ◯ TREATMENT/RECOVERY HOUSE
- ◯ JAIL
- ◯ COVID EMERGENCY HOUSING
- ◯ OTHER:________

90 Have you used any non-injection drugs in the last 2 months?

- ◯ YES
- ◯ NO

105 Have you used any injection drugs in the last 2 months?

- ◯ YES
- ◯ NO

Indicate if, or how often you have done the following since March 2020 ?

		NEVER (0% OF THE TIME)	OCCASIONALLY (25% OF THE TIME OR LESS)	SOMETIMES (26-74% OF THE TIME)	USUALLY (75% OF THE TIME OR MORE)	ALWAYS (100% OF THE TIME)
136	Worn a mask in public places	◯	◯	◯	◯	◯
137	Practiced physical distancing in public places	◯	◯	◯	◯	◯
138	Avoided crowded places/gatherings	◯	◯	◯	◯	◯
139	Avoided common greetings (such as a handshake or a hug)	◯	◯	◯	◯	◯
140	Limited contact with people at higher risk (e.g., an elderly relative)	◯	◯	◯	◯	◯

150 What are the sources of information you use to make your decision about getting the COVID-19 vaccine? (READ; Check all that apply)

- ☐ FAMILY MEMBERS
- ☐ FRIENDS
- ☐ OTHER PEOPLE WHO USE DRUGS (PEERS, DEALERS, ETC
- ☐ MY MD
- ☐ NURSES/ SOCIAL WORKER/ PHARMACIST
- ☐ HARM REDUCTION WORKER (OPS, SIF, NEP)
- ☐ VCH OUTREACH WORKERS
- ☐ MEDIA LIKE NEWSPAPERS, TV, RADIO
- ☐ SOCIAL MEDIA LIKE FACEBOOK OR INSTA
- ☐ YOUTUBE
- ☐ OTHER (SPECIFY BELOW)
- ☐ OTHER (SPECIFY BELOW)

(Still ask if not wanting to get vax)

157	If you do not plan to get the COVID-19 vaccine, what are the reasons you do not plan to get it? (Do not read; Select all that apply)	☐ I'M ALLERGIC TO VACCINES<br>☐ I DON'T LIKE NEEDLES<br>☐ I'M NOT CONCERNED ABOUT GETTING REALLY SICK FRO COVID-19<br>☐ I'M CONCERNED ABOUT THE SIDE EFFECTS OF THE VACC<br>☐ I DON'T THINK VACCINES WORK WELL<br>☐ I DON'T TRUST THE VACCINE WILL BE SAFE<br>☐ I DON'T TRUST DOCTORS<br>☐ I DON'T TRUST THE GOVERNMENT<br>☐ I DON'T BELIEVE THE COVID-19 IS AS BAD AS SOME PEOPLE SAY IT IS<br>☐ I DON'T KNOW ENOUGH ABOUT HOW WELL A COVID-19 VACCINE WORKS<br>☐ I ALREADY HAD COVID-19<br>☐ I HAVE BEEN POORLY TREATED BY DOCTORS OR OTHER HEALTHCARE WORKERS<br>☐ OTHER (SPECIFY BELOW)
159	If you did get the COVID-19 vaccine, or plan on getting it, what are the reasons why?	☐ I AM WORRIED ABOUT GETTING COVID-19<br>☐ I DO NOT WANT TO PASS COVID TO MY FRIENDS, FAMILY OR PEERS<br>☐ I AM CONFIDENT IT IS SAFE<br>☐ I AM CONFIDENT IT IS EFFECTIVE<br>☐ A DOCTOR/NURSE/HEALTHCARE WORKER TOLD ME TO<br>☐ A FRIEND/FAMILY MEMBER/PEER TOLD ME TO<br>☐ I HAVE A HEALTH CONDITION THAT COULD CAUSE A SERIOUS CASE OF COVID-19<br>☐ I DO NOT LIKE MASKING/SOCIAL DISTANCING OR OTHER COVID-19 PROTECTION MEASURES<br>☐ I AM NOT ABLE TO MAKE/SOCIAL DISTANCE OR EMPLOY OTHER COVID-19 PROTECTIVE MEASURES<br>☐ OTHER (SPECIFY BELOW)

Notes: column 1 refers to the item number in the interviewer administered questionnaire, column 2 includes the questions asked of the participants and column 3 includes the response options provided by the participants.
